# Investigation of multiple adsorption mechanisms for efficient removal of ofloxacin from water using lignin-based adsorbents

**DOI:** 10.1038/s41598-018-37206-1

**Published:** 2019-01-24

**Authors:** Boqiang Gao, Pei Li, Ran Yang, Aimin Li, Hu Yang

**Affiliations:** State Key Laboratory of Pollution Control and Resource Reuse, School of the Environment, Nanjing University, Nanjing, 210023 P. R. China

## Abstract

Two series of lignin (LN)-based adsorbents, namely, cross-linked lignin (LNEs) with different crosslinking densities and carboxymethyl cross-linked lignin (LNECs) with various degrees of carboxymethyl substitution, were prepared to remove ofloxacin (OFL), a popular fluoroquinolone (FQ) antibiotic, from water. LNEs and LNECs exhibited satisfactory performance in OFL adsorption. Both of them had high adsorption capacity (the maximum contribution of 0.828 mmol/g), good anti-interference to some inorganic salts, and efficient regeneration and reuse performance. The crosslinking density and degree of carboxymethyl substitution strongly affected the content and distribution of oxygen-containing groups in these LN-based adsorbents, which played important roles in OFL adsorption. The pH dependencies of the adsorption performance of LNEs and LNECs indicated the involvement of multiple adsorption mechanisms, including hydrogen bond, electrostatic attraction, π-π electron–donor–acceptor interactions, and negative charge-assisted hydrogen bond. Different mechanisms were dominant under various pH levels, in a near neutral pH, the synergistic effect of electrostatic attraction and π-π interaction allows LINEs and LINECs to reach maximum adsorption capacity. Five FQs with similar structures and their two sub structural analogs were compared in terms of adsorption behavior and electrostatic potential by density functional theory using quantum chemical calculation. FQs with secondary amino groups and low π electron cloud density readily bound to LN-based adsorbents. Hence, LNEs and LNECs were efficient and environment-friendly adsorbents.

## Introduction

Over the past few decades, fluoroquinolones (FQs) have gained increasing attention for treatment of broad-spectrum bacterial infections in humans and animals^1^. In China, two of the top five antibiotics used in humans in 2013 were FQs, namely, ofloxacin (OFL) and norfloxacin^2^; in animal husbandry, more than 4,000 tons of both enrofloxacin and ciprofloxacin used as veterinary antibiotics are consumed every year^3^. FQs are discharged to the natural environment mainly through wastewater produced by the pharmaceutical industry and solid waste generated by human beings and livestock. Existing sewage treatment facilities have low processing efficiency^4^. This leads to the long-term presence of FQs in the environment, which may cause increased bacterial resistance, affecting the activities of aquatic organisms and severe damage to the ecological environment^5^.

Many technologies have been developed for removal of FQs from water; such technologies include adsorption^6^, advanced oxidation process^7^ (AOPs), biodegradation^4^, and membrane separation^8^. Biodegradation and membrane separation (especially ultrafiltration membranes) have low removal efficiencies^4^. AOPs employ complex operating conditions, and the toxicity of their degradation products remains unknown^9^. Thus, adsorption is considered one of the most effective technologies for pollutant removal due to its simple operation and low processing costs^10^. Adsorbents are the key in efficient adsorption of contaminants. Many adsorbents have been used to remove FQs; these adsorbents include activated carbon^11^, porous resins^12^, carbon nanotubes^6^, graphene^13^, and biochar^14–16^.

Natural organic polymeric adsorbents, including lignin (LN), cellulose, starch, chitosan, and their derivatives, have received much attention due to their wide resource and environment friendliness^17–21^. Among them, LN is the second largest natural polymer and largely exists in black liquor in paper manufacturing^22^. In fact, LN and its derivatives after some chemical modifications have been widely applied in water treatment, which could be used as adsorbents, flocculants, and scale inhibitors^23,24^. As effective adsorbents due to their having abundant oxygen-containing functional groups, lignin-based materials were employed to remove various contaminants in water such as heavy metals^25,26^ and cationic dyes^27,28^ in previous study. Besides, LN still contains aromatic ring structure^29^, which is typical in molecules of many organic materials, such as FQs. However, limited research is available with regard to the use of LN-based adsorbents for removal of FQs; moreover, the corresponding adsorption mechanisms using LN as adsorbent have been insufficiently studied due to the complicated structural characteristics of LN and contaminants^15,16,26^.

In this work, two series of LN-based adsorbents, namely, cross-linked lignin (LNEs) with different crosslinking densities and carboxymethyl cross-linked lignin (LNECs) with various degrees of carboxymethyl substitution, were designed and prepared. The former was prepared using epichlorohydrin (EPI) as crosslinking agent to increase its chemical stability, and the latter was prepared using chloroacetic acid (CA) as etherification agent. OFL, a popular FQ antibiotic, was selected as the target contaminant. The fundamental OFL adsorption behavior of LNEs and LNECs, including the pH effect, adsorption equilibrium, available interference of inorganic and organic additives, and recycling use, were investigated, respectively. Multiple adsorption mechanisms were discussed in detail based on the structural effects of the adsorbent and adsorbate. The effects of the content and distribution of oxygen-containing groups on LN-based adsorbents due to their various crosslinking densities and degrees of carboxymethyl substitution as well as pH dependencies of different OFL species in water have been investigated. Moreover, the adsorption properties of the four other FQs with similar structures to OFL [norfloxacin (NOR), ciprofloxacin (CIP), enrofloxacin (ENR), and fleroxacin (FLE)] and their two sub structural analogs [flumequine (FLU) and 1-(2-Fluorophenyl) (FPP)] as molecular probes were compared. Their electrostatic potentials (ESP) were estimated by density functional theory (DFT) using quantum chemical calculation to further explore the adsorption mechanism.

## Results and Discussion

### Characterization of LNEs and LNECs

**Figure 1:**
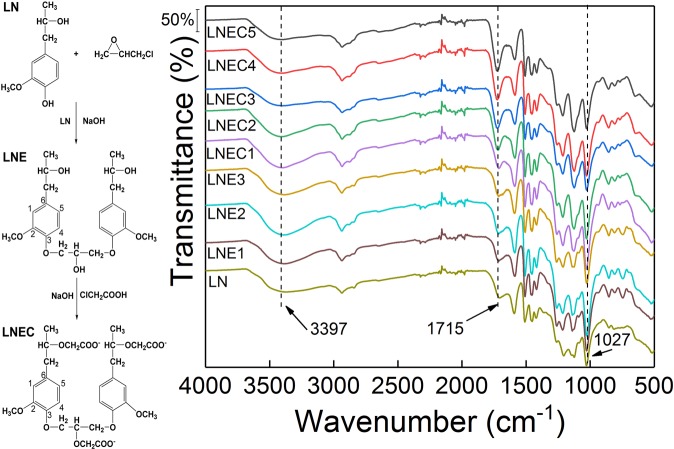
FTIR spectra of LN, LNEs and LNECs, and the inset is the samples preparation process, in which the basic structural unit of LN, coniferyl alcohol, was chosen as a representative of this complex polymer.

Three LNEs with different crosslinking densities and five LNECs with various degrees of carboxymethyl substitution were obtained according to the inset of Fig. 1 by controlling the fed amounts of crosslinking agent (EPI) and etherification agent (CA), respectively, which were described in detail in the experimental section. After chemically crosslinking, the stabilities of those lignin-based materials in water was significantly improved during a wide pH range from 3.0 to 11.0. (Supporting Information Fig. S1). The surface morphologies of LNEs and LNECs samples turned rougher and more compact owing to modifications in comparison with smooth surface of pure LN (Supporting Information Fig. S2).

FTIR spectra of LN, LNEs and LNECs were measured and shown in Fig. 1. Accordingly, three characteristic peaks at 1588, 1507 and 1417 cm^−1^ were caused by the stretching vibration of benzene ring in LN^30^. The characteristic peaks at 3397, 1715, and 1027 cm^−1^ were ascribed to three oxygen-containing groups on LN, i.e. –O-H, –C=O of carboxylic compounds^31^, and –C-O of secondary alcohols and ethers^32^, respectively. The FTIR spectrum of LN was quite similar to that of LNEs, which indicated that the crosslinking reaction could not significantly change the lignin structure and functional groups. In addition, the intensity of the characteristic peak at 1715 cm^−1^ for five LNECs clearly increased owing to the successful introduction of carboxymethyl groups onto LN after etherification.

For more detailed characterization, isoelectric point (pH_zpc_) and the contents of various oxygen-containing groups on those LNEs and LNECs were determined and listed in Table 1. The pH_zpc_ of LNEs slightly increased because of the decrease of the content of carboxyl groups with the increase of the fed amount of crosslinking agent from LNE1 to LNE3. However, the content of negatively charged carboxyl groups increased with the increase of the fed amount of CA from LNEC1 to LNEC5, causing the coinstantaneous decrease of their pH_zpc_ from 4.60 to 3.94, which has the same trend as the results reported by other researchers^33^. In addition, the contents of lactone in LNEs and LNECs had almost no change, but those of phenolic hydroxyl groups decreased significantly after the crosslinking and etherification. Moreover, the increased content of carboxyl group was greater than the decreased content of phenolic hydroxyl group in etherification (Table 1). Those findings indicate that the crosslinking and etherification reactions might mainly take place on the phenolic hydroxyl groups and the ortho position of phenyl ring due to their higher activities^33,34^.

**Table 1:** Preparation receipt, pH_zpc_, the contents of various oxygen-containing functional groups, and the adsorption capacities at optimal pH conditions of different LNEs and LNECs samples.

Lignin-based samples	Amounts of EPI (mL)	Amounts of CA (g)	*q*_e_^a^ (mmol/g)	pH_zpc_	Oxygen-containing functional groups (mmol/g)				${R}_{adj}^{2}$ for the linear simulation between *q*_e_ and the amount of various functional groups		
					Carboxyl	Phenolic hydroxyl	Lactone	Acidic^b^	Carboxyl	C + P^c^	Acidic^b^
LNE1	1.0	—	0.478	4.52	0.470	0.402	0.375	1.247	0.719	0.765	0.697
LNE2	2.0	—	0.426	4.60	0.405	0.285	0.403	1.093			
LNE3	4.0	—	0.407	4.62	0.305	0.138	0.400	0.843			
LNEC1	2.0	0.2	0.517	4.56	0.562	0.253	0.322	1.137	0.942	0.916	0.863
LNEC2	2.0	1.0	0.638	4.44	0.988	0.222	0.327	1.537			
LNEC3	2.0	2.0	0.675	4.38	1.092	0.195	0.390	1.677			
LNEC4	2.0	3.0	0.727	4.17	1.230	0.170	0.413	1.813			
LNEC5	2.0	4.0	0.828	3.94	1.372	0.135	0.347	1.853			

^a^Adsorption capacity at the optimal pH levels: pH of 6.0 for LNEs and 7.0 for LNECs.

^b^The sum of carboxyl, phenolic hydroxyl, and lactone groups.

^c^The sum of carboxyl and phenolic hydroxyl groups.

### Effect of pH

**Figure 2:**
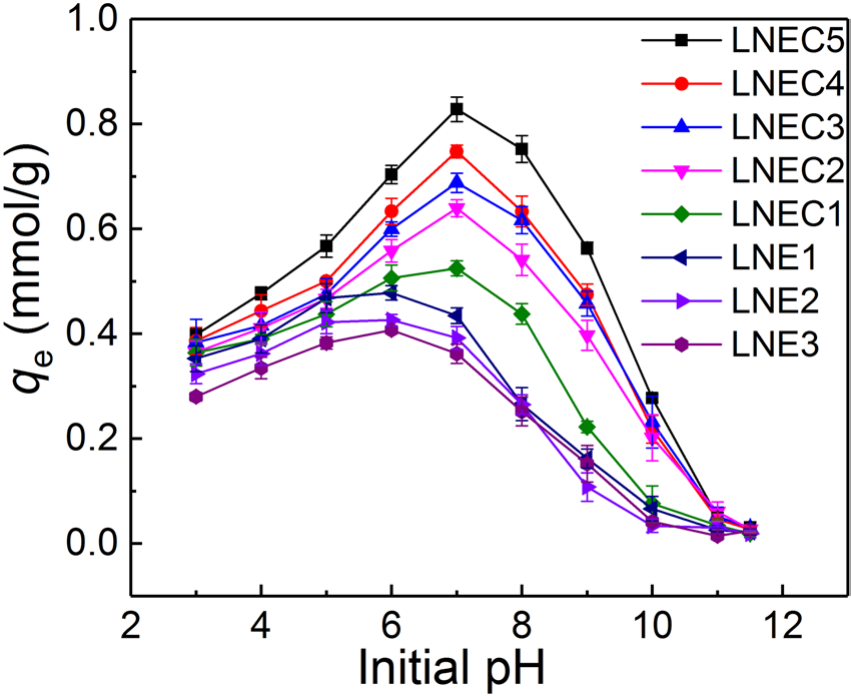
The effects of initial pH on the adsorption capacities of LNEs and LNECs for OFL removal (*C*_0,OFL_ = 0.2 mmol/L).

The two series of LN-based adsorbents were employed to remove OFL from water. The pH effects were tested first owing to the significant influence of pH on adsorption behaviors, as shown in Fig. 2. Accordingly, LNEs and LNECs exhibited very similar pH dependence in each series. With the increase of pH, OFL uptakes increased at the beginning, reached a maximal value at initial solution pH of about 6.0 for LNEs but 7.0 for LNECs, and then decreased rapidly. The optimal adsorption capacities of LNEs and LNECs were higher than those of most of previously reported adsorbents (Supporting Information Table S1), indicating that these LN-based adsorbents had an advantageous effect in adsorption of OFL. As for the upward-climax-downward variation tendencies in the pH dependence of OFL uptakes by those two series of LN-based adsorbents, it was ascribed to the fact that pH would substantially change the surface charge properties of both adsorbents and adsorbates, and various adsorption mechanisms were involved to further affect the adsorption behaviors, which would be discussed in detail in the following section.

Moreover, the adsorption capacities of LNEs and LNECs dropped to near zero at initial pH of around 11.5 (Fig. 2). Thus, 0.001 mol/L aqueous NaOH solution was selected as the desorption eluent to regenerate the LN-based adsorbents after saturated adsorption. Figure S3 in Supporting Information shows that the adsorption capacities of LNEC5 and LNE2 were reduced by only about 20% after five adsorption–desorption recycles. It suggested that the LN-based materials exhibited fine reusability, which was quite satisfactory for practical applications. In addition, the effects of various inorganic salts (NaCl, KCl, and Na_2_SO_4_) indicated that the OFL uptakes of those LN-based adsorbents decreased with the increase of salt concentrations (Supporting Information Fig. S4) owing to electrostatic shielding and competitive adsorption effects^35^. As for the coexisted organic matter, humic acid (HA), OFL uptakes of those LN-based adsorbents slightly increased at the beginning and then decreased with increasing the HA concentration (Supporting Information Fig. S5a). The improved OFL uptakes at a low HA concentration (<10 mg/L) might be due to a bridging effect linked lignin-based adsorbents and FQs^36^, since HA and lignin-based adsorbents have the similar molecular structures. At a higher HA concentration, this bridging effect was broken because of enhanced electrostatic repulsion between HA and lignin-based adsorbents; besides, Fig. S5b in Supporting Information indicated an interaction existed between HA and OFL^37^, resulting in its competitive adsorption of OFL with LNEC5 and LNE2. In short, the inhibition rates of the three salts and HA on adsorption capacities of those lignin-based adsorbents were below 30% in the entire measured salt and HA concentration ranges, indicating that LNECs and LNEs possessed good anti-interference to these coexisted matters.

### Effect of Oxygen-Containing Groups

LN contains many oxygen-containing groups such as carboxyl, phenolic hydroxyl, and lactone, which could act as adsorption active sites but had different influences on OFL adsorption owing to their various affinities with OFL. The contents of the functional groups would change after the crosslinking and etherification reactions as discussed in previous section, resulting in different adsorption capacities of LNEs and LNECs. Table 1 lists the maximal OFL uptakes of those LN-based adsorbents at their optimal pH levels.

Based on Table 1, the maximal OFL uptakes of LNEs decreased with the decrease of the contents of both carboxyl and phenolic hydroxyl groups owing to the reduction of adsorption active sites. For LNECs, although the content of phenolic hydroxyl groups decreased with the increase of the degree of carboxyl substitution, the sum of carboxyl and phenolic hydroxyl groups still increased from LNEC1 to LNEC5, resulting in improved adsorption capacities. Moreover, the molar stoichiometric ratio of total oxygen-containing groups to OFL uptakes was much higher than 1:1 (Table 1), indicating that many functional groups on the LN-based adsorbents could not be involved in this adsorption process. This finding may be caused by two facts. One was the steric effects of OFL having large molecular size. The other was very weak affinity of certain oxygen-containing group to OFL, resulting in little contribution to the adsorption. OFL contains various functional groups and is an amphoteric substance which has two *pK*_a_ values (*pK*_a1_ = 5.98, *pKa*_2_ = 8.00, Table 2). Accordingly, at pH of about 6.0–7.0, OFL mainly showed cationic feature. For three oxygen-containing groups on LN, lactone was very difficult to ionize and had little electrostatic interaction with OFL, whereas carboxyl group (*pK*_a_ ≈ 5.00) of LN was more easily ionized than phenolic hydroxyl group^25,38^ (*pK*_a_ ≈ 7.00), causing stronger electrostatic attractions of carboxyl group to OFL. These facts might result in the contributions of these three functional groups to OFL adsorption followed the order of carboxyl > phenolic hydroxyl > lactone groups in this situation.

**Table 2:** The physicochemical properties (including molecular structure, pK_a_, log K_ow_, and molecular weight) of five FQs and two molecular probe species used in this work.

Adsorbates	Molecular structure	log K_ow_	Molecular weight	Adsorbates	Molecular structure	log K_ow_	Molecular weight
OFL pK_a1_ = 5.98^1^ pK_a2_ = 8.00^1^		−0.39^7^	361	CIP pK_a1_ = 6.14^1^ pK_a2_ = 8.85^1^		0.28^7^	331
NOR pK_a1_ = 6.27^1^ pK_a2_ = 8.71^1^		0.46^7^	319	ENR pK_a1_ = 6.20^1^ pK_a2_ = 8.13^1^		0.7^56^	359
FLE pK_a1_ = 5.46^1^ pK_a2_ = 8.00^1^			369	CBZ /		2.45^50^	236
FPP pK_a1_ = 4.49^55^ pK_a2_ = 8.63^55^			180	FLU pK_a_ = 6.29^55^		2.70^56^	261

In addition, the linear relationships between those oxygen-containing groups and the OFL uptakes of the LN-based adsorbents are shown in Supporting Information Fig. S6, and correlation coefficients (*R*^2^_adj_) are listed in Table 1. All *R*^2^s were not high, around 0.7–0.9, indicating that other effects such as π-π interactions might have contributions to OFL adsorption in addition to the oxygen-containing functional groups. More details about the adsorption mechanisms would be discussed in the following section.

### Adsorption Mechanism at Different pH Regions

The adsorption performance is strongly related to the micro-structural natures of both adsorbent and adsorbate in water. On the basis of the analysis of the molecular structures of two LN-based adsorbents and OFL shown in the inset of Fig. 1 and Table 2, OFL with various functional groups and aromatic structure has two *pK*_*a*_s and possesses three species (OFL^+^, OFL^0^, and OFL^−^) in aqueous solution owing to its amphoteric feature^1^. Similarly, the LN-based adsorbents contained various oxygen-containing groups and phenylpropane structures with various pH_zpc_s (Table 1), resulting in various surface charges under different pH conditions. Therefore, the available involved interactions between the LN-based adsorbents and OFL mainly included electrostatic attraction [Eq. (1)], hydrogen bond (H-bond) [Eq. (2)], π-π electron-donor-acceptor interaction [Eq. (3)], and hydrophobic interactions^15,26,39^. In this study, the contribution of hydrophobic interactions to adsorption may be low because both OFL (logK_ow_ = −0.39) and LN-based adsorbents (rich in carboxyl and hydroxyl groups) have good hydrophilicity, which would be discussed in detail in the following section.

1 $$L{N}^{-}+OF{L}^{+}\to L{N}^{-}\cdot OF{L}^{+}\,\,\,\,{\rm{electrostatic}}\,{\rm{attraction}}$$

2 $$LN\,-\,O\,-\,H+OF{L}^{0}\,-\,N\to LN\,-\,O\,-\,H\,\cdots \,N\,-\,OF{L}^{0}\,\,\,\,{\rm{H}} \mbox{-} {\rm{bond}}$$

3 $$LN+OFL\to LN\,\cdots \,OFL\,\,\,\,{\rm{\pi }} \mbox{-} {\rm{\pi }}\,{\rm{interaction}}$$

**Figure 3:**
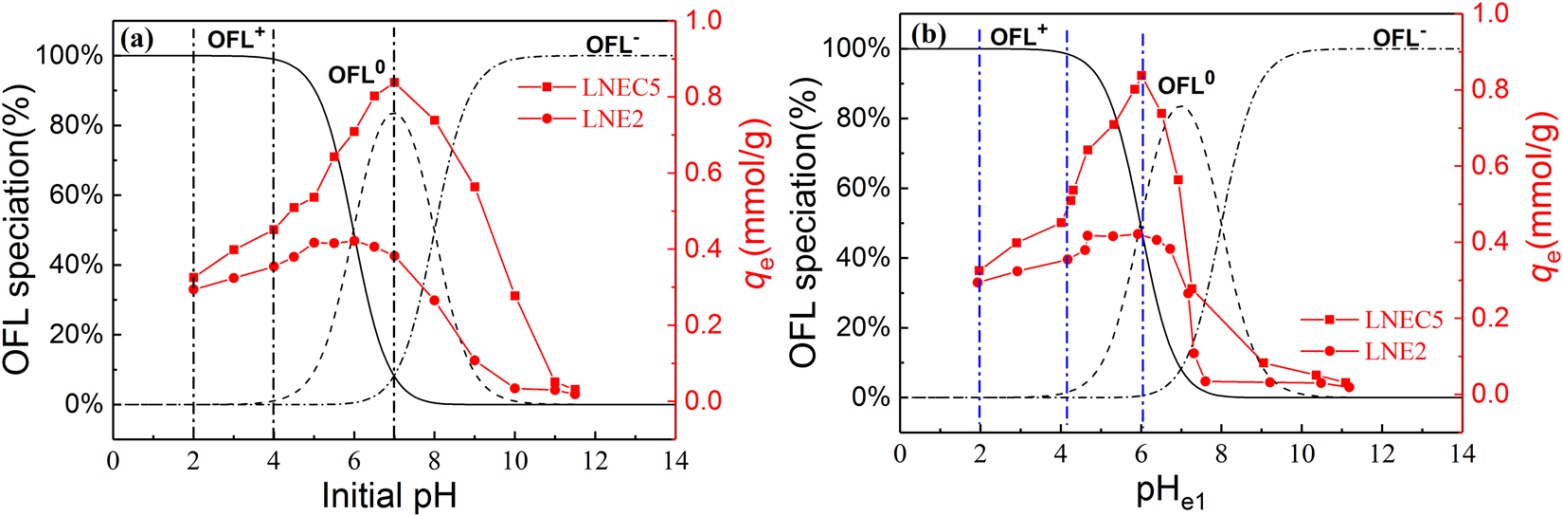
Effects of (**a**) initial pH and (**b**) pH_e1_ on adsorption capacities of LNE2 and LNEC5 (*C*_0,OFL_ = 0.2 mmol/L), and species distributions of OFL at different pH values.

**Figure 4:**
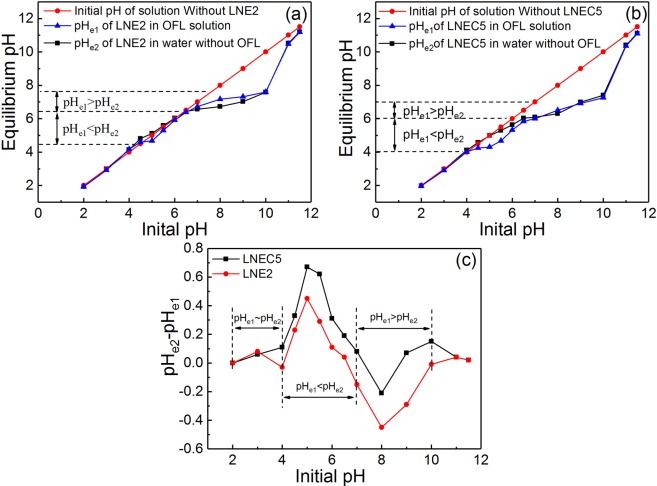
The initial solution pH dependences of pH_e1_ and pH_e2_ for (**a**) LNE2 and (**b**) LNEC5 at the initial OFL concentration of 0.2 mmol/L; (**c**) The initial solution pH dependences of the difference between pH_e2_ and pH_e1_ of LNE2 and LNEC5 according to (**a**,**b**), respectively.

However, the structures and charge properties of both LN-based adsorbents and OFL would be changed by pH, causing their various adsorption behaviors and mechanisms at different pH levels. Based on Fig. 2, LNE2 and LNEC5 were selected as the representatives to further explore the adsorption mechanisms at various pH regions because LNEs and LNECs showed very similar pH dependence of OFL adsorption in each series of LN-based adsorbents. According to the variation tendencies of the adsorption capacities of LNE2 and LNEC5 with pH (Fig. 3), there are two points of inflexion appeared in their adsorption capacity- pH curves at pH about 4.0 and 7.0, respectively. The discontinuous variation in adsorption capacity implied different adsorption mechanisms involved in different pH regions. Therefore, three initial pH regions were roughly divided as acidic (2.0–4.0), weak acidic and neutral (4.0–7.0), and alkaline (7.0–11.5), as shown in Fig. 3a. For better discussion of the pH effects on OFL adsorption and the involved adsorption mechanisms, the corresponding adsorption equilibrium pH (pH_e1_) range, 2.0–4.2, 4.2–6.0, and 6.0–11.5, is given in Fig. 3b and used as a reference. Moreover, the equilibrium pH (pH_e2_) of LNE2 and LNEC5 dissolved in water without OFL but at different initial pH values was recorded. The initial solution pH dependences of pH_e1_ and pH_e2_ are shown in Fig. 4a,b. The difference between pH_e2_ and pH_e1_ of LNE2 and LNEC5 according to Fig. 4a,b at various initial pH values is shown in Fig. 4c.

#### Acidic Region

At initial pH range of 2.0–4.0, i.e., pH_e1_ 2.0–4.2, the content of cationic OFL species (OFL^+^) was dominant (Fig. 3). In contrast, most carboxyl and phenolic hydroxyl groups of LNE2 and LNEC5, with the pH_zpc_ of 4.60 and 3.94, respectively, were protonized and even exhibited weak cationic features. Thus, electrostatic attractions and π-π interaction were weak, but H-bond between OFL and LN-based adsorbents may be significant. Moreover, according to Fig. 4c, the difference between pH_e2_ and pH_e1_ of LNE2 and LNEC5 was quite small at the pH range of 2.0–4.0, further confirming that H-bond was dominant in this situation because of minimal influence of H-bond on the change of H^+^ concentration.

The FTIR spectra of LNE2 and LNEC5 before and after adsorption of OFL at initial pH 3.0 are shown in Supporting Information Fig. S7. The characteristic absorption peak of conjugated carboxyl groups appeared at 1620 cm^−1^ after the adsorption of OFL on LNE2 and LNEC5^31^, indicating that OFL was adsorbed on the LN-based adsorbents. Moreover, the characteristic peaks at 1714 and 1728 cm^−1^ corresponding to carbonyl groups on LNE2 and LNEC5 moved to 1710 and 1716 cm^−1^, respectively. This may be caused by the formation of H-bonds between the carboxyl group of LN-based adsorbents and the amine group of OFL, resulting in a decrease in energy and a red shift^40,41^. The red shift of LNEC5 was more apparent, resulting from more H-bonds formed in LNEC5.

Moreover, with the increase of pH, the adsorption capacities of LNE2 and LNEC5 slowly increased owing to the reduction of protonization effect. The adsorption capacity of LNEC5 increased more than that of LNE2 (Fig. 3) because LNEC5 with a lower pH_zpc_ contained more carboxyl groups and was easier to deprotonate.

#### Weak Acidic and Neutral Region

In the range of initial pH at 4.0–7.0 (pH_e1_ from 4.2–6.0), the adsorption capacities of LNE2 and LNEC5 further increased, and those of LNEC5 were more evident. Based on Fig. 3b, the content of OFL^+^ decreased from 99% to 48%, but that of neutral OFL increased in this pH range; whereas the deprotonation effect of two LN-based adsorbents was both enhanced causing their negative surface charges increased. Electrostatic attractions were significantly improved. Moreover, the adsorption capacity of LNEC5 continuously and rapidly increased until to the initial pH of 7.0, whereas that of LNE2 changed insignificantly but slowly with increasing pH, indicating that LNEC5 exhibited stronger electrostatic attractions with OFL because it contained more carboxyl groups than LNE2.

To further explore the adsorption mechanisms of LNE2 and LNEC5 changed with the pH, the difference of pH_e1_ and pH_e2_ was compared (Fig. 4c). Accordingly, pH_e1_ was smaller than pH_e2_ at the initial pH range 4.0–7.0, indicating that some protons were released in the adsorption process owing to the ionization effect of oxygen-containing groups promoted through electrostatic attractions^14,39,42^, That is, the carboxyl group on the lignin-based adsorbent might undergo a proton substitution by the cationic species of OFL, causing the decrease of the solution pH, as shown in Eq. (4):

4 $$LN\,-\,COOH+OF{L}^{+}\to LN\,-\,COO\,-\,OFL+{H}^{+}$$

The difference between pH_e1_ and pH_e2_ of LNEC5 was more apparent than that of LNE2 owing to higher adsorption capacity of LNEC5 and release of more protons. Moreover, the difference between pH_e1_ and pH_e2_ of both LN-based adsorbents reached the maximum when the initial pH was about 5.0 (Fig. 4c), which was ascribed to the strongest electrostatic interactions for having suitable contents of cationic OFL and anionic oxygen-containing groups on LN. The results are consistent with previous studies that the cation exchange reaches a maximum at a certain pH between the pH_zpc_ of the adsorbent and the pKa (acid) of the adsorbate^14,43^. In addition, the maximal OFL uptakes of LNE2 and LNEC5 were not at the initial pH of 5.0 (Fig. 2), indicating that other interactions such as π-π interaction were also involved in this adsorption in addition to electrostatic attractions in this pH range. OFL and LN-based materials both contained aromatic structures, and some neutral OFL form and protonized oxygen-containing groups on LN-based adsorbents coexisted. These may result in π-π interaction between them.

#### Alkaline Region

At initial pH range of 7.0–11.5 (pH_e1_ from 6.0 to 11.5), the adsorption capacities of LNE2 and LNEC5 rapidly decreased (Fig. 3) and even reached near zero at initial pH higher than 10.0. In this pH range, the content of OFL^+^ further reduced, but that of OFL^−^ gradually appeared; the negative surface charges of both LN-based adsorbents were further enhanced, resulting in increased electrostatic repulsion between them. Thus, the effects of electrostatic attractions, H-bond, and π-π interactions were all weakened. However, the adsorption capacities of LNE2 and LNEC5 were still maintained a relative high level at the initial pH range between 7.0 and 10.0 (pH_e1_ from 6.0 to 8.0). According to Fig. 4c, pH_e1_ was higher than pH_e2_ at this initial pH range of 7.0–10.0, indicating that OH^−^ was released into the solution or protons were adsorbed during this adsorption process.

Negative charge-assisted H-bond^39,44,45^ (CAHB) was a special H-bond, which was easily generated when the H-bond donor and H-bond acceptor had similar pK_a_ values^46^ (ΔpK_a_ = pK_a_,_H-bond donor_ − pK_a,H-bond acceptor_ ≤ ~4.00). In this work, the ΔpK_a_ between the H-bond donor (OFL-N-H) and the H-bond acceptor (LN-O^−^) was approximately equal to 1.0 (pK_a_,_H-bond donor_ = 8.00, pK_a,H-bond acceptor_ ≈ 7.00). Thus, CAHB may be involved in adsorption of OFL by LN-based adsorbents in alkaline conditions. This process is shown in Eqs (5 and 6):

5 $$OF{L}^{-}\,-\,N+{H}_{{\rm{2}}}O\to OF{L}^{0}\,-\,N\,-\,H+O{H}^{-}$$

6 $$LN\,-\,{O}^{-}+OF{L}^{0}\,-\,N\,-\,H\to LN\,-\,{O}^{-}\,\cdots \,H\,-\,N\,-\,OF{L}^{0}$$

The anionic species of OFL took a proton from water to obtain neutral OFL form, which could easily form H-bond with the deprotonated phenolic hydroxyl groups of LN-based adsorbents. OH^−^ was simultaneously released into the solution, so the pH of equilibrium adsorption increased^39^. This CAHB effect of LNE2 was stronger than that of LNECs, resulting from a higher difference between pH_e2_ and pH_e1_ in LNE2 observed in Fig. 4c. This finding could be due to the numerous phenolic hydroxyl groups in LNE2. Moreover, the phenolic hydroxyl group of LN has a low ΔpK_a_ value with OFL and has a strong CAHB effect. However, CAHB would be significantly inhibited in strong alkaline conditions^47^ because the reaction described in Eq. (5) would be depressed at higher concentration of OH^−^. As a result, the adsorption capacities of LNE2 and LNEC5 were reduced to near zero at initial pH higher than 10.0.

### Isothermal Adsorption Analysis

**Figure 5:**
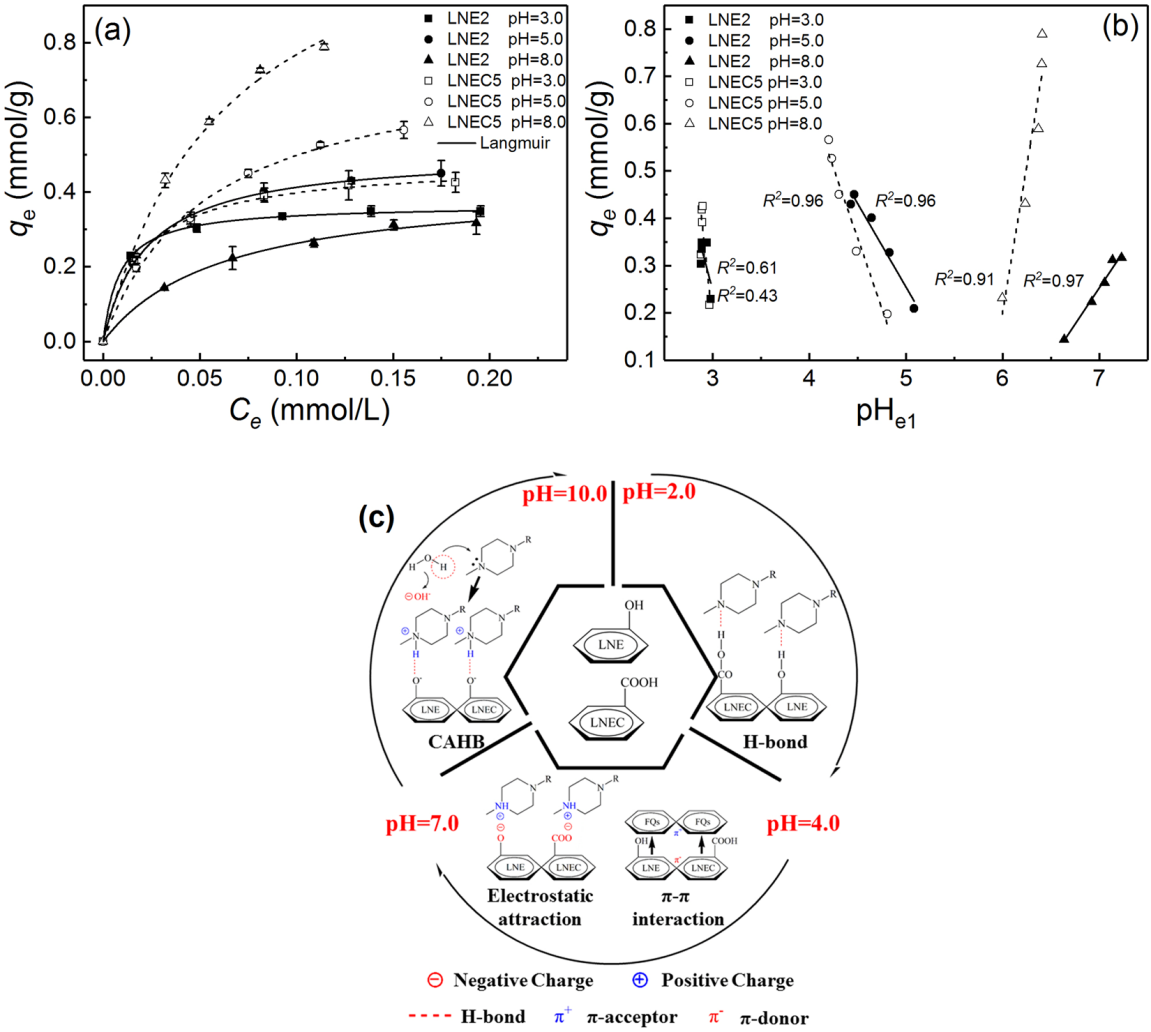
(**a**) Adsorption isotherms of LNE2 and LNEC5 at initial pH of 3.0, 5.0, and 8.0, respectively, with initial OFL concentrations of 0.05–0.25 mmol/L; (**b**) the pH_e1_ dependences of adsorption capacities of LNE2 and LNEC5 at various pH levels according to (**a**,**c**) the possible adsorption mechanisms at different pH conditions.

Moreover, the adsorption isotherms of LNE2 and LNEC5 for OFL removal at initial pH of 3.0, 5.0, and 8.0 in different pH regions, i.e., acidic, weak acidic, and alkaline regions, were studied and shown in Fig. 5a to well understand the possible interactions between adsorbate and adsorbent. These obtained adsorption equilibrium data were analyzed based on Langmuir^48^ and Freundlich model^49^ to further explore their adsorption mechanisms; the results are listed in Supporting Information Table S2, and the detailed model descriptions are exhibited in Supporting Information Text S1. Based on the *R*^2^_adj_s, the adsorption isotherms of LNE2 and LNEC5 all closely followed Langmuir model, which indicated that the adsorption behaviors of these LN-based adsorbents in entire measured pH range were a monolayer chemical adsorption. This finding confirmed that hydrophobic interactions could not affect the OFL adsorption while the other three ones, i.e., electrostatic attraction, H-bond, and π-π interactions, might be involved.

The relationships between the equilibrium pH (pH_e1_) and the adsorption capacities of two LN-based adsorbents at the initial pH values of 3.0, 5.0, and 8.0 under different initial OFL concentrations are shown in Fig. 5b. At the initial pH of 3.0, the pH_e1_ decreased very slightly after reaching adsorption equilibrium; moreover, ${R}_{adj}^{2}$ s of LNE2 and LNEC5 were both maintained at a much lower level (0.43 for LNE2 and 0.61 for LNEC5), indicating that the adsorption process in this pH condition was independent on pH. This finding further confirmed that H-bond was the dominant effect for OFL adsorption by LN-based adsorbents in acidic region because H-bond was less effective on pH in adsorption. The initial pH was 5.0, the pH_e1_s of LNE2 and LNEC5 both linearly decreased with the increase of OFL uptakes and their ${R}_{adj}^{2}$ s were higher (0.96 for both LNE2 and LNEC5). This result showed that more protons were promoted to release into water when more OFL was adsorbed onto these LN-based adsorbents owing to electrostatic attractions. In contrast, at the initial of pH 8.0, the pH_e1_ increased linearly with the increase of adsorption of OFL, but the ${R}_{adj}^{2}$ s were still both higher (0.97 for LNE2 and 0.91 for LNEC5) because of the effects of CAHB as discussed above. The isothermal adsorptions were fully consistent with the previous pH-dependent results and further confirmed various adsorption mechanisms involved in different pH regions, as summarized in Fig. 5c.

### Analysis of Molecular Structures

To further investigate the effects of molecular structure on the OFL adsorption by LN-based adsorbents, the adsorption of two sub structural analogs as molecular probes (FLU and FPP) and four FQs with similar structures to OFL (NOR, CIP, ENR, and FLE), of which detailed physicochemical properties are shown in Table 2, were carried out. Their isotherms and detailed analysis with OFL together are shown in Supporting Information Fig. S8 and Table S3. The Langmuir model fitted all adsorption isotherms better, indicating that seven substances obeyed similar adsorption manners owing to their similar molecular structures.

**Figure 6:**
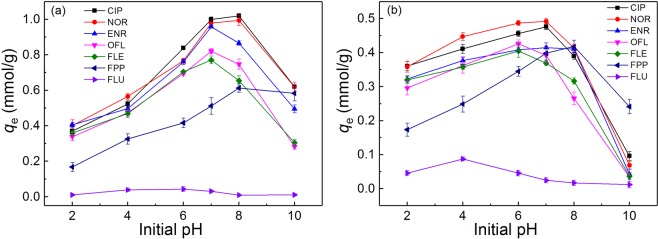
Effect of pH on adsorption capacities of (**a**) LNE2 and (**b**) LNEC5 for removal of various FQs and two molecular probes [*C*_0_ = 0.2 mmol/L, except to the initial concentration of FLU is 0.05 mmol/L due to its low solubility (logk_ow_ = 2.7)].

Moreover, Fig. 6 shows the pH dependencies of adsorption of the seven substances. Interestingly, LNE2 and LNEC5 both exhibited far different adsorption capacities for two molecular probes, i.e., the FLU uptakes were much lower than FPP ones in the entire measured pH range. This finding indicated two facts. One was that the adsorption of OFL might mainly take place on the nitrogen-containing heterocyclic part. In addition to the electrostatic repulsion effects between LN-based adsorbents and FLU owing to the carboxyl groups both contained, hydrophobic effects for this adsorption were weak, because FLU with a high logK_ow_ value approximate 2.70 had strong hydrophobicity but very low adsorption capacity. Moreover, the pH effect of CBZ (logK_ow_ = 2.45) adsorption, another pharmaceutical with a higher hydrophobicity and almost no ionization in aqueous solution^50^, was also measured and shown in Supporting Information Fig. S9. Accordingly, the adsorption capacities of LN-based adsorbents for CBZ were similar to those for FLU and were lower than 0.1 mmol/g, further confirming the hydrophobic effect was ineffectual in this system.

The adsorption capacities of five different FQs with similar structures (Fig. 6) exhibited similar upward–climax–downward variation tendencies with the increase of pH, but the optimal pH values corresponding to maximal adsorption capacities were shifted to left or right owing to their different pK_a_ values (Table 2). The adsorption capacities of LNE2 and LNEC5 for the five FQs followed the order NOR ≈ CIP > ENR > OFL > FLE. NOR and CIP showed higher affinities to LN-based adsorbents. The piperazine ring of NOR and CIP, including the molecular probe of FPP, were all linked to the secondary amine, whereas those of ENR, OFL, and FLE were linked to the tertiary amine. According to the pK_a2_ value of NOR (pK_a2_ = 8.71), CIP (pK_a2_ = 8.85), ENR (pK_a2_ = 8.13), OFL (pK_a2_ = 8.00), and FLE (pK_a2_ = 8.00) listed in Table 2, the secondary amine group had higher capability to obtain protons and show cationic forms than the tertiary amine group^51^, so the adsorption efficiencies of NOR and CIP were higher because of the stronger electrostatic attractions with LN-based adsorbents. However, the adsorption capacities of LNE2 and LNEC5 for FPP were lower than those of NOR and CIP, indicating that electrostatic attraction was not the only mechanism for promoting the adsorption.

### Molecular Simulation

**Figure 7:**
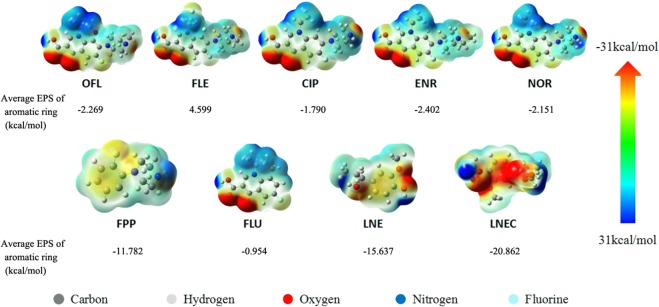
The electronic cloud density (ECD) of five FQs, two molecular probes, LNE and LENC. The inset numbers are the average EPS of the entire aromatic ring of various compounds, and the average EPS of benzene is −13.594 kcal/mol.

In addition to electrostatic attractions, the π-π interaction was also one of the main mechanisms for the adsorption of aromatic ring antibiotics under weak acidic and neutral conditions as mentioned above^39^. The different groups linked to the aromatic rings caused the aromatic ring compound to enhance or weaken the electron cloud density (ECD) and become π-donor or π-receptor. DFT was applied to calculate the electrostatic potentials (ESP) of the five FQs, two molecular probes, and two LN-based adsorbents in this work. Figure 7 shows that the ECD in the red region was dense, and the blue region was scarce^52^, exhibiting that ECDs of two LN-based adsorbents were higher than those of seven antibacterials.

The average EPS on the aromatic rings of each compounds including each carbon atom on aromatic rings was estimated using Multiwfn software^53^ as shown in Supporting Information Table S4 and Fig. 7. Using the average ESP of benzene(−13.594 kcal/mol) as a reference, the order of the average ESPs of various compounds was FLE > FLU > CIP > NOR > OFL > ENR > FPP > benzene > LNE > LNEC. This finding indicated that the ECDs of five FQs and two molecular probes were weakened owing to the comprehensive effects of both induction and conjugation originated from those various linked atoms and groups such as fluorine and piperazine ring^52^, which could act as π-acceptors; whereas those of LN-based adsorbents were enhanced because of the electron-donating effects of the linked hydroxyl and carboxymethyl groups, which could be used as π-donor in this system. Therefore, π-π interactions may occur between the LN-based adsorbents as the π-donor and the FQs as the π-acceptor. Moreover, ECD of LNEC was higher than that of LNE owing to more oxygen-containing groups on LNEC5 (Table 1). Thus, FQs with low π electron cloud density were more ready to bind with aforementioned LN-based adsorbents.

However, the order of the average EPS on the aromatic rings of seven antibacterials was not consistent with that of their adsorption capacities (Fig. 6). This finding indicates multiple adsorption mechanisms were involved in this situation. Comparing the five FQs, FLE has the largest EPS value, but its amine group has the weakest capability to obtain protons (pK_a2_ = 8.0). According to the adsorption capacities (Fig. 6), the contribution of electrostatic attraction to adsorption was greater than π-π interaction. This finding was because π-π interactions as a kind of intermolecular forces was relatively weak, which would be significantly influenced by other stronger effects such as electrostatic interactions. The adsorption of CIP, NOR, and ENR was thus greater than that of OFL and FLE. However, NOR and ENR have different pK_a2_ values, but their maximum adsorption capacities and EPS were similar (Fig. 6) owing to the enhanced π-π interactions in near neutral conditions promoted by electrostatic attractions, which could effectively shorten the distance of aromatic rings between adsorbent and adsorbate^40,54^. In both acidic and alkaline conditions, the electrostatic repulsions between OFL and LN-based adsorbents hindered the formation of π-π interactions. Therefore, the maximal adsorption capacities of LNEs and LNECs, which appeared at near neutral conditions were the result of the synergistic effects of electrostatic attraction and π-π interactions.

## Conclusions

In this study, two series of LN-based adsorbents, LNEs and LNECs, were obtained and exhibited good performance in adsorption of OFL. After LN was cross-linked using EPI, the mechanical properties and chemical stability of LNEs were enhanced evidently; whereas further carboxymethyl modified LNECs showed improved adsorption capacities. Various adsorption mechanisms were involved at different pH levels. At acidic levels (initial pH of 2.0–4.0), H-bond mainly formed between –COOH or -OH of LN-based adsorbents, and –N-C of OFL was dominant. At weak acidic and neutral conditions (initial pH of 4.0–7.0), the synergistic effect of electrostatic attraction and π-π interaction allows LINEs and LINECs to reach maximum adsorption capacity. In π-π interactions, LN-based adsorbents acted as π-donor, whereas OFL served as π-acceptor. At alkaline levels (initial pH of 7.0–11.5), a special H-bond, charge-assisted H-bond, had a significant contribution to OFL adsorption. For more specific investigations at molecular level, four FQs with similar structures to OFL and their two sub structural analogs as molecular probes were studied and compared in their various adsorption behaviors and electrostatic potentials. FQs with secondary amino groups and low π electron cloud density were easier to bind with the LN-based adsorbents. Therefore, LNEs and LNECs can be used as efficient and environmental-friendly adsorbents for removal of FQs. This study provides theoretical guidance for the large-scale production of lignin-based adsorbents using low-cost industrial grade lignin in the future.

## Materials and Methods

### Chemicals and Materials

LN and FPP (>98 wt%) were purchased from TCI Chemical Industry Development Co., Ltd. (Shanghai, China). OFL (>98 wt%), NOR (>98 wt%), CIP (>98 wt%), ENR (>98 wt%), FLE (>98 wt%), FLU (>98 wt%), and carbamazepine (CBZ, >98 wt%) were obtained from Aladdin Chemistry Co. Ltd. (Shanghai, China). The detailed physicochemical properties of the five FQs and the two molecular probes are listed in Table 2 ^1,7,50,55,56^. EPI was purchased from Shanghai Lingfeng Co., Ltd. CA, acetonitrile (HPLC grade), acetic acid (HPLC grade), NaOH, HCl, NaCl, KCl, and other reagents used in this study were all analytical grade and obtained from Sinopharm Chemical Reagent Co. Ltd. Milli-Q water (18.2 MΩ·cm) was used for high-performance liquid chromatography (HPLC) and all solutions were stored at 277 K prior to use.

### Preparation of LNEs and LNECs

The synthesis procedure of LNE and LNEC is shown in the inset of Fig. 1 brief, in which the basic structural unit of LN, coniferyl alcohol, was chosen as a representative of this complex polymer^29^. 4 g of LN was added to 30 mL of distilled water with an initial pH of 10 adjusted by adding 0.1 mol/L aqueous NaOH solution. The mixture was alkalized for 1 h at 333 K in water bath under stirring and added drop wise with a desired amount of EPI. The reaction was kept for 6 h to obtain LNE. A series of LNEs with different crosslinking densities, named LNE1, LNE2, and LNE3, respectively, was obtained by the same method but added with different amounts of crosslinking agents (Table 1).

LNE2 was selected to further prepare LNEC. Two g of purified LNE2 and a known amount of NaOH were fully mixed with 90 mL of ethanol. After a full alkalization for 1 h at 343 K in water bath under stirring, a desired amount of CA was drop wise added into the mixture within 30 min. The reaction was kept for 2 h to obtain LNEC. A series of LNECs with different degrees of carboxymethyl substitution, referred to as LNEC1, LNEC2, LNEC3, LNEC4, and LNEC5, was obtained using the same method but feeding different amounts of CA according to Table 1.

### Characterization of LNEs and LNECs

The FTIR spectra of the LN-based adsorbents were recorded using a Fourier transform infrared spectrometer (TENSOR 27, Bruker Co.) in the wavenumber range of 500–4000 cm^−1^ with a resolution of 0.4 cm^−1^. The surface microstructures of LNEs and LNECs were observed directly by an environmental scanning electron microscope (ESEM) (SSX-550, Shimadzu Co.). The point of zero charge of pH (pHzpc) of the LN-based adsorbents was determined by acid-base titration using an automatic titration system^57^ (T50, Mettler Toledo). The contents of oxygen-containing functional groups on the surface of LNEs and LNECs were determined by Boehm titration method^58^ using NaOH, Na_2_CO_3_, and NaHCO_3_ standard solutions for obtaining the contents of phenolic hydroxyl, lactone, and carboxyl groups on the LN-based adsorbents. The stability of LN, LNE2, and LNEC5 in water has been tested. Approximately 0.15 g of dried lignin-based material was dispersed in 900 mL of water at different pH conditions adjusted by using 0.1 mol/L HCl or 0.1 mol/L NaOH aqueous solutions. The dissolved lignin in water was determined using an ultraviolet spectrophotometer (UV2600A) at the wavelength of 280 nm.

The ESPs of five FQs (OFL, NOR, CIP, ENR, and FLE), two molecular probes (FLU and FPP), and two LN-based adsorbents (LNE and LENC) were theoretically optimized by DFT with Gaussian 09 program at b3lyp/6-31 g* level.

### Batch Experiments for Adsorption

OFL was selected as the main target contaminant in this work. The pH effects of various LNEs and LNECs on OFL adsorption were measured. For better comparison and further investigation of adsorption mechanisms, LNE2 and LNEC5 were then selected as the representatives of two series of LN-based adsorbents in further measurements including adsorption isotherms at various pH levels, effects of some salts, and reusability experiments. The pH effects and adsorption isotherms of LNE2 and LNEC5 for adsorption of other four FQs (NOR, CIP, ENR, and FLE) with similar structure to OFL, and their two sub structural analogs (FLU and FPP) as molecular probes were all studied. Moreover, the pH dependence of CBZ adsorption, a pharmaceutical with a higher hydrophobicity than OFL and almost no ionization in aqueous solution^50^, was conducted for comparison. All batch experiments for adsorption experiments were carried out in the absence of light to avoid its available interference. All experiments were measured in three runs, and the result was the average of the triplicate with the relative error lower than 5%.

### Effect of pH

The effect of initial solution pH was conducted at 298 K. The measured initial pH range was 2.0–11.5 adjusted by adding 0.1 mol/L aqueous HCl or NaOH solution. Approximately 5.0 mg of dried adsorbent was dispersed in 30 mL of pollutant solution with the initial concentration of 0.2 mmol/L at various initial pH values under continuous stirring at 180 rpm in an incubator shaker for 6 h. The equilibrium pH (pH_e1_) after adsorption was recorded using a Delta320 pH meter (Mettler–Toledo, Switzerland). For better comparison, the equilibrium pH (pH_e2_) of the LN-based adsorbents in water without OFL but under different initial pH conditions was also measured.

The concentrations of various contaminants were detected by an Agilent HPLC Series 1200 equipped with An Agilent^®^ Zorba^TM^ C18 column (4.6 × 250 mm × mm, 5 μm) coupled to a diode-array detector (DAD) detector after filtration using 0.45 μm filter. Isocratic elution was at 1.0 mL/min, injection volume was 10 μL, and the column temperature was 298 K. Specific analytical conditions for various contaminants are described in Supporting Information Table S5.

By calculating the change of pollutant concentration in the adsorption process, equilibrium adsorption capacity (*q*_e_, mmol/g) can be determined, and the calculation equation is represented as follows:

7 $${q}_{e}=\frac{({C}_{0}-{C}_{e})V}{m}$$

where *C*_0_ and *C*_e_ (mmol/L) are the concentrations of the solution before adsorption and after reaching adsorption equilibrium, respectively; *V* (L) is the volume of solution; and *m* (mg) is the dried weight of the adsorbents.

### Adsorption Equilibrium Study

Adsorption isotherms of LNE2 and LNEC5 for OFL adsorption were carried out at 298 K and different initial pH conditions, i.e., 3.0, 5.0, and 8.0, respectively. Moreover, the isothermal adsorptions of OFL, NOR, CIP, ENR, FLE, FLU, and FPP by LNE2 and LNEC5 were performed and compared at 298 K and initial pH of 7.0. The initial concentrations of various pollutants ranged from 0.05–0.25 mmol/L. The adsorption capacity was estimated based on Eq. (7).

### Effects of additives

The adsorption performance of LNEC5 and LNE2 in the presence of inorganic and organic additives (i.e., NaCl, KCl, Na_2_SO_4,_ and HA) at 298 K and initial pH of 8.0 was studied. About 5.0 mg of adsorbent was immersed in 30 mL of OFL solution with the initial concentrations of 0.2 mmol/L, which contained different amounts of inorganic salt with the concentration range of 0–10 mmol/L or HA with that of 0–100 mg/L. A similar analysis method was employed to detect the final OFL concentrations once adsorption equilibrium was achieved.

### Reusability Experiments

The LNEC5 and LNE2 after saturated adsorption of OFL were regenerated using 100 mL of a 0.001 mol/L aqueous NaOH solution stirring for 24 h at room temperature. LNEC5 and LNE2 after desorption were collected by filtration, washed with distilled water, dried in an oven, and reused in the next cycle of adsorption experiments. The adsorption–desorption experiments were conducted for five cycles.

## Supplementary information

Supporting information
