# Supplementary material for: Investigation of multiple adsorption mechanisms for efficient removal of ofloxacin from water using lignin-based adsorbents
Source: Sci Rep. 2019 Jan 24;9:637. doi: 10.1038/s41598-018-37206-1 (PMC6346052; doi:10.1038/s41598-018-37206-1)
Supplement: Supplementary file 1 — Supporting information [file 41598_2018_37206_MOESM1_ESM.docx]

**Supporting Information Cover Sheet**

**Investigation of multiple adsorption mechanisms for efficient removal of ofloxacin from water using lignin-based adsorbents***

Boqiang Gao, Pei Li, Ran Yang, Aimin Li, Hu Yang**

State Key Laboratory of Pollution Control and Resource Reuse, School of the Environment, Nanjing University, Nanjing 210023, P. R. China

Number of pages: 20

Number of tables: 5

Number of figures: 9

Number of texts: 1

* Supported by the Natural Science Foundation of China (grant nos. 51778279 and 51438008), the Natural Science Foundation of Jiangsu Province of China (grant no. BK20161405), and Six Talent Peaks Project in Jiangsu Province of China (grant no. 2015-JNHB-003).

** Corresponding author. Tel & Fax: 86-25-89681272, E-mail: yanghu@nju.edu.cn

**Table of Contents**

| **Index** | **Captions** | **Page** |
| --- | --- | --- |
| **Table S1** | Adsorption capacities of different adsorbents for removal of OFL reported in recent years. | S4 |
| **Table S2** | The fitting parameters of the Langmuir and Freundlich for the adsorption isotherms of LNE2 and LNEC5 at various initial pHs of 3.0, 5.0, and 8.0, respectively. | S5 |
| **Table S3** | Adsorption isotherms of LNE2 and LNEC5 for adsorption of five FQs and two molecular probe species at initial pH of 7.0 (*C*_0_=0.2 mmol/L). | S6 |
| **Table S4** | The average surface electrostatic potentials of the entire aromatic ring of various compounds and those of each carbon atom on the aromatic ring. | S7 |
| **Table S5** | Chromatographic conditions and detector parameters for various compounds. | S8 |
| **Figure S1** | The stability of LN, LNE2, and LNEC5 in water at different equilibrium pHs (0.15 g of dried lignin-based material was dispersed in 900 mL of water at different pH conditions). | S9 |
| **Figure S2** | Environmental scanning electron microscope images of (a) lignin, (b) LNE1, (c) LNE2, (d) LNE3, (e) LNEC1, (f) LNEC2, (g) LNEC3, (h) LNEC4, and (i) LNEC5. | S10 |
| **Figure S3** | Regeneration and reuse performance of LNEC5 and LNE2 at initial pH of 7.0 (*C*_0,OFL_ = 0.2 mmol/L). | S11 |
| **Figure S4** | Effects of inorganic concentration on OFL removal by LNE2 and LNEC5 (*C*_0,OFL_ = 0.2 mmol/L). | S12 |
| **Figure S5** | (a) Effects of humic acid (HA) on adsorption capacities of LNE2 and LNEC5 for removal of OFL (C_0,OFL_=0.2 mmol/L). (b) The actual OFL concentrations in various HA aqueous solutions with different contents. | S13 |
| **Figure S6** | The effect of carboxyl and hydroxyl groups contents on adsorption capacities of LNEs and LNECs. | S14 |
| **Figure S7** | The FTIR of LNE2 and LNEC5 before and after adsorption of OFL at initial pH 3.0. | S15 |
| **Figure S8** | Adsorption isotherms of (a) LNE2 and (b) LNEC5 for removal of FQs at initial pH of 7.0. | S16 |
| **Figure S9** | The effects of pH on the adsorption capacities of LNE2 and LNEC5 for carbamazepine (CBZ) removal. | S17 |
| **Text S1** | Adsorption isothermal models. | S18 |
| **Reference** |  | S19-S20 |

**Table S1.** Adsorption capacities of different adsorbents for removal of OFL reported in recent years.

| Adsorbent | Adsorption capacity (mmol/g) | Source |
| --- | --- | --- |
| cassava residue-derived biochar | 0.008 | R1 |
| Sediments | 0.11 | R2 |
| Mesoporous silica | 0.32 | R3 |
| Titanate nanobelts | 0.41 | R4 |
| Activated carbon  (barley straw) | 1.12 | R5 |
| rice-husk and  wood-chip biochars | 0.022 | R6 |
| nanoparticles | 0.14 | R7 |
| zeolite imidazolate framework | 0.35 | R8 |
| sawdust | 0.047 | R9 |
| Hydroxyapatite | 0.083 | R10 |
| Carbon nanotubes | 0.43 | R11 |
| lotus stalk-based activated carbon | 0.923 | R12 |
| LNE2 | 0.426 | This work |
| LNEC5 | 0.828 | This work |

**Table S2.** The fitting parameters of the Langmuir and Freundlich models for the adsorption isotherms of LNE2 and LNEC5 at various initial pHs of 3.0, 5.0, and 8.0, respectively.

| **Adsorbents** | **pH** | ***q*_exp_**  **(mmol/g)** | **Langmuir model** | | | **Freundlich model** | | |
| --- | --- | --- | --- | --- | --- | --- | --- | --- |
|  |  |  | ***q*_m,cal_ (mmol/g)** | ***K*_L_**  **(L**/**mmol)** | $\boldsymbol{R}_{\boldsymbol{adj}}^{\boldsymbol{2}}$ | ***K_F_*** | ***n*** | $\boldsymbol{R}_{\boldsymbol{adj}}^{\boldsymbol{2}}$ |
| LNE2 | 3.0 | 0.348 | 0.365 | 114.708 | 0.999 | 0.469 | 6.392 | 0.991 |
| LNE2 | 5.0 | 0.429 | 0.505 | 44.370 | 0.998 | 0.777 | 3.441 | 0.988 |
| LNE2 | 8.0 | 0.312 | 0.424 | 16.203 | 0.996 | 0.656 | 2.405 | 0.989 |
| LNEC5 | 3.0 | 0.418 | 0.479 | 49.169 | 0.998 | 0.702 | 3.817 | 0.982 |
| LNEC5 | 5.0 | 0.526 | 0.768 | 18.434 | 0.997 | 1.375 | 2.198 | 0.990 |
| LNEC5 | 8.0 | 0.726 | 1.269 | 15.359 | 0.994 | 2.752 | 1.811 | 0.977 |

**Table S3.** Adsorption isotherms of LNE2 and LNEC5 for adsorption of five FQs and two molecular probe species at initial pH of 7.0 (*C*_0_=0.2mmol/L).

| **Adsorbents** | **Adsorbate** | ***q_exp_***  **(mmol/g)** | **Langmuir model** | | | | **Freundlich model** | | |
| --- | --- | --- | --- | --- | --- | --- | --- | --- | --- |
|  |  |  | ***q_m,cal_* (mmol/g)** | ***K_L_***  **(L**/**mmol)** | ***R_L_*×10^2^** | $\boldsymbol{R}_{\boldsymbol{adj}}^{\boldsymbol{2}}$ | ***K_F_*** | ***n*** | $\boldsymbol{R}_{\boldsymbol{adj}}^{\boldsymbol{2}}$ |
| LNE2 | NOR | 0.529 | 0.564 | 160.563 | 3.987 | 0.999 | 0.806 | 5.400 | 0.972 |
|  | CIP | 0.470 | 0.506 | 152.974 | 6.136 | 0.998 | 0.684 | 6.015 | 0.969 |
|  | ENR | 0.432 | 0.439 | 248.767 | 3.864 | 0.990 | 0.573 | 7.303 | 0.987 |
|  | OFL | 0.382 | 0.400 | 296.383 | 3.264 | 0.999 | 0.485 | 9.565 | 0.993 |
|  | FPP | 0.410 | 0.461 | 44.267 | 10.149 | 0.995 | 0.693 | 3.544 | 0.986 |
|  | FLE | 0.331 | 0.329 | 336.802 | 2.883 | 0.997 | 0.389 | 10.849 | 0.998 |
|  | FLU | 0.114 | 0.194 | 7.128 | 48.329 | 0.961 | 0.275 | 1.825 | 0.941 |
| LNEC5 | CIP | 1.004 | 1.743 | 44.915 | 8.177 | 0.985 | 5.775 | 1.943 | 0.957 |
|  | NOR | 0.994 | 1.560 | 53.285 | 6.983 | 0.996 | 4.789 | 2.103 | 0.973 |
|  | ENR | 0.983 | 1.263 | 93.727 | 4.093 | 0.995 | 2.955 | 2.831 | 0.955 |
|  | OFL | 0.777 | 0.913 | 80.709 | 4.722 | 0.992 | 1.735 | 3.245 | 0.987 |
|  | FLE | 0.774 | 0.862 | 94.090 | 4.078 | 0.997 | 1.541 | 3.542 | 0.970 |
|  | FPP | 0.424 | 0.468 | 76.959 | 11.500 | 0.998 | 0.678 | 4.410 | 0.990 |
|  | FLU | 0.077 | 0.138 | 6.268 | 61.469 | 0.983 | 0.192 | 1.758 | 0.971 |

**Table S4.** The average surface electrostatic potentials of the entire aromatic ring of various compounds and those of each carbon atom on the aromatic ring.

| Compounds | Average surface electrostatic potentials (kcal/mol) | | | | | | |
| --- | --- | --- | --- | --- | --- | --- | --- |
|  | C1^a^ | C2 | C3 | C4 | C5 | C6 | Aromatic ring |
| FLE | 6.632 | 9.403 | 9.642 | 1.000 | -2.110 | 4.439 | 4.599 |
| FLU | 0.172 | 3.456 | 4.274 | -3.335 | -6.912 | -1.559 | -0.954 |
| CIP | 1.226 | 0.225 | 1.707 | -4.993 | -6.985 | -0.437 | -1.790 |
| NOR | 0.463 | 0.121 | 1.890 | -5.476 | -7.565 | -1.052 | -2.151 |
| OFL | -0.283 | 2.528 | 3.324 | -5.211 | -8.739 | -3.156 | -2.269 |
| ENR | 0.390 | -0.419 | 1.162 | -5.497 | -7.562 | -1.040 | -2.402 |
| FPP | -10.616 | -11.552 | -13.354 | -13.688 | -12.123 | -9.087 | -11.782 |
| Benzene | -13.573 | -13.630 | -13.576 | -13.596 | -13.602 | -13.589 | -13.594 |
| LNE | -19.297 | -14.536 | -13.597 | -13.729 | -15.464 | -19.190 | -15.637 |
| LNEC | -25.228 | -22.970 | -20.750 | -19.170 | -18.031 | -22.525 | -20.862 |

^a^: The order of carbons on benzene ring of various FQs and lignin-based adsorbents are shown in Fig. 1 and Table 2, respectively.

**Table S5.** Chromatographic conditions and detector parameters for various compounds.

| Group | Compound | Mobile phase  composed of A and B (V:V) | UV wavelength (nm) |
| --- | --- | --- | --- |
| Fluoroquinolones | OFL | 85:15* | 285 |
|  | CIP | 85:15* | 285 |
|  | ENR | 35:65* | 285 |
|  | NOR | 85:15* | 285 |
|  | FLE | 85:15* | 285 |
|  |  |  |  |
| Molecular probe species | FLU | 50:50* | 232 |
|  | FPP | 85:15* | 232 |
|  |  |  |  |
|  | CBZ | 20:80** | 276 |

*: Mobile phase A was composed of HPLC grade 0.8% (V/V) acetic acid, and mobile phase B was HPLC grade acetonitrile;

**: Mobile phase A was HPLC grade 0.1mol/L formic acid solution, and mobile phase B was HPLC grade acetonitrile.

**Figure S1.** The stability of LN, LNE2, and LNEC5 in water at different equilibrium pHs (0.15 g of dried lignin-based material was dispersed in 900 mL of water at different pH conditions).


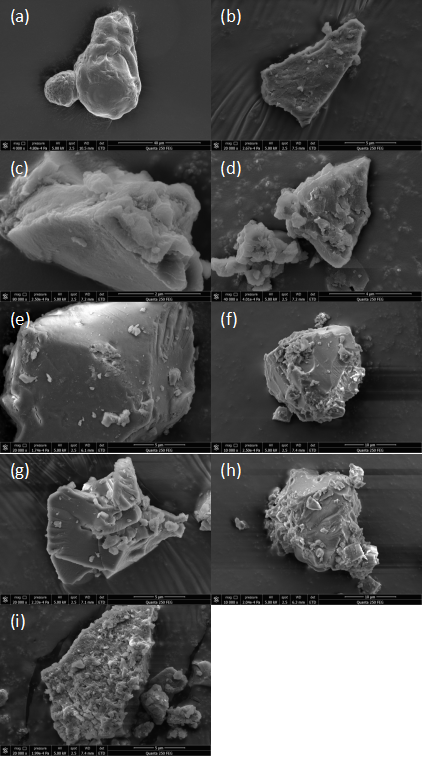


**Figure S2.** Environmental scanning electron microscope (ESEM) images of (a) lignin, (b) LNE1, (c) LNE2, (d) LNE3, (e) LNEC1, (f) LNEC2, (g) LNEC3, (h) LNEC4, and (i) LNEC5.

 **Figure S3.** Regeneration and reuse performance of LNEC5 and LNE2 at initial pH of 7.0 (*C*_0,OFL_ = 0.2 mmol/L).

**Figure S4.** Effects of inorganic concentration on OFL removal by LNE2 and LNEC5 (*C*_0,OFL_ = 0.2 mmol/L).

**Figure S5.** (a) Effects of humic acid (HA) on adsorption capacities of LNE2 and LNEC5 for removal of OFL (C_0,OFL_=0.2 mmol/L). (b) The actual OFL concentrations in various HA aqueous solutions with different contents.

**Figure S6.** The effect of carboxyl and hydroxyl groups contents on adsorption capacities of LNEs and LNECs. P+C: the sum of carboxyl and phenolic hydroxyl groups. Acidic: the sum of carboxyl, phenolic hydroxyl, and lactone groups.

**Figure S7.** The FTIR of LNE2 and LNEC5 before and after adsorption of OFL at initial pH 3.0.

 **Figure S8.** Adsorption isotherms of (a) LNE2 and (b) LNEC5 for removal of FQs at initial pH of 7.0.

**Figure S9.** The effects of pH on the adsorption capacities of LNE2 and LNEC5 for carbamazepine (CBZ) removal.

**Text S1** Adsorption isothermal models.

Langmuir model^R13^ can be expressed as follows,

$q_{e}=\frac{q_{m}K_{L}C_{e}}{1+K_{L}C_{e}}$ (S1)

Where *q_e_* (mmol/g) is the amount of metal ions adsorbed at equilibrium, *C_e_* (mmol/L) is the equilibrium concentration of metal ion, *q_m_* (mmol/g) is the adsorption capacity when the adsorbent is fully covered, and *K_L_* (L/mmol) is the Langmuir adsorption constant.

Freundlich model^R14^ is another frequently applied isotherm model which equation is expressed as follows,

$q_{e}=K_{F}C_{e}^{1/n}$ (S2)

Where *K_F_* is the Freundlich isotherm constant, and *n* (dimensionless) is the heterogeneity factor, respectively.

*R_L_*^R15^ is a dimensionless constant that can reflect essential characteristics of the model.

$R_{L}=\frac{1}{1+K_{L}C_{0}}$ (S3)

Where *C_0_* (mmol/L) is the initial concentration of OFL, *K_L_* is the adsorption affinity parameter of Langmuir model. Adsorption is favorable when 0<*R_L_*<1, and the smaller the value of *R_L_*, the stronger the attraction between the adsorbent and the adsorbate.

**References**

R1. Huang, P., Ge, C.J., Feng, D., Yu, H.M., Luo, J.W., Li, J.T., Strong, P.J., Sarmah, A.K., Bolan, N.S., Wang, H.L. Effects of metal ions and pH on ofloxacin sorption to cassava residue-derived biochar. Sci. Total Environ. **616-617**, 1384-1391 (2018).

R2. Wang, P., Zhang, D., Zhang, H., Li, H., Ghosh, Saikat., Pan, B. Impact of concentration and species of sulfamethoxazole and ofloxacin on their adsorption kinetics on sediments. Chemosphere. **175**, 123-129 (2017).

R3. Jin, T., Yuan, W.H., Xue, Y.J., Wei, H., Zhang, C.Y., Li, K.B. Co-modified MCM-41 as an effective adsorbent for levofloxacin removal from aqueous solution: optimization of process parameters, isotherm, and thermodynamic studies. Environ. Sci. Pollutr. **24**, 5238-5248 (2017).

R4. Li, W.Y., Wang, J.R., He, G.J., Yu, L, Noor, Nuruzzaman, Sun, Y.G., Zhou, X.Y., Hu, J.Q., Parkin, Ivan P. Enhanced adsorption capacity of ultralong hydrogen titanate nanobelts for antibiotics. J. Mater. Chem. A. **5**, 4352-4358 (2017).

R5. Yan, B., Niu, C.H. Modeling and site energy distribution analysis of levofloxacin sorption by biosorbents. Chem. Eng. J. **307**, 631-642 (2017).

R6. Yi, S.Z., Gao, B., Sun, Y.Y., Wu, J.C., Shi, X.Q., Wu, B.J., Hu, X. Removal of levofloxacin from aqueous solution using rice-husk and wood-chip biochars. Chemosphere **150**, 694-701 (2018).

R7. Wu, H., Shi, Y.T., Guo, X.Z., Zhao, S.L., Du, J.L., Jia, H.P., He, L.N., Du, L.M. Determination and removal of sulfonamides and quinolones from environmental water samples using magnetic adsorbents. J Sep Sci. **39**, 4398-4407 (2016).

R8. Zhou, Q., Zhu, L.H., Xia, X.L., Tang, H.Q. The water - resistant zeolite imidazolate framework 67 is a viable solid phase sorbent for fluoroquinolones while efficiently excluding macromolecules. Microchim Acta. **183**, 1839-1846 (2016).

R9. Qureshi, T., Memon, N., Memon, S.Q., Ashraf, M.A. Decontamination of ofloxacin: optimization of removal process onto sawdust using response surface methodology. Desalin. Water. Treat. **57**, 221-229 (2016).

R10. Tang, W.L., Zhao, J.C., Sha, B.J., Liu, H. Adsorption and Drug Release Based on beta-Cyclodextrin-Grafted Hydroxyapatite Composite. J. Appl. Polym. Sci. **127**, 2803-2808 (2013).

R11. Peng, H.B., Pan, B., Wu, M., Liu, Y., Zhang, D., Xing, B.S. Adsorption of ofloxacin and norfloxacin on carbon nanotubes: hydrophobicity- and structure-controlled process. J Hazard Mater. **233-234**, 89-96 (2012).

R12. Liu, W.F., Zhang, J., Zhang, C.L., Ren, L. Sorption of norfloxacin by lotus stalk-based activated carbon and iron-doped activated alumina: Mechanisms, isotherms and kinetics. Chem. Eng. J. **171**, 431-438 (2011).

R13. Langmuir, I. The adsorption of gases on plane surfaces of glass, mica and platinum. J. Am. Chem. Soc. **40**, 1361-1403 (1918).

R14. Freundlich, H. Über die adsorption in lösungen. Zeitschrift für Physikalische **57**, 385-470 (1906).

R15. Li, K., Li, P., Cai, J., Xiao, S.J., Yang, H., Li, A.M. Efficient adsorption of both methyl orange and chromium from their aqueous mixtures using a quaternary ammonium salt modified chitosan magnetic composite adsorbent. Chemosphere. **154**, 310-318 (2016).
